# Role of nitric oxide in optic nerve head blood flow regulation while experimental increase of ocular perfusion pressure

**DOI:** 10.1186/1471-2210-11-S2-A47

**Published:** 2011-09-05

**Authors:** Reinhard Told, Doreen Schmidl, Michael Lasta, Agnes Boltz, Berthold Pemp, Semira Kaya, Gerhard Garhöfer, Gabriele Fuchsjäger-Mayrl, Leopold Schmetterer

**Affiliations:** 1 Department of Clinical Pharmacology, Medical University of Vienna, 1090 Vienna, Austria; 2 Department of Ophthalmology and Optometry, Medical University of Vienna, 1090 Vienna, Austria; 3 Center for Medical Physics and Biomedical Engineering , Medical University of Vienna, 1090 Vienna, Austria

## Background

The involvement of nitric oxide (NO) in choroidal blood flow regulation during experimental increase of ocular perfusion pressure (OPP) has been shown in previous studies. It is also known that during isometric exercise, the inhibition of NO synthase (NOS) leads to a rightward shift of pressure-flow curves. In this study the influence of inhibited NOS on optic nerve head (ONH) blood flow during isometric exercise was investigated.

## Methods

A randomized, double-blinded, placebo-controlled, three-way crossover design was chosen for the present study. In order to increase systemic perfusion pressure during application of either a NOS inhibitor (L-NMMA), an α-receptor agonist (phenylephrine) or placebo, 18 healthy subjects were asked to squat for 6 minutes. Laser Doppler flowmetry (LDF) was used for continuous assessment of ONH blood flow and OPP was calculated as 2/3 mean arterial pressure minus intraocular pressure (IOP).

## Results

L-NMMA and phenylephrine both significantly increased OPP at rest (p < 0.001 vs. baseline). However, only L-NMMA significantly decreased ONH blood flow at rest compared to baseline (p = 0.02). While isometric exercise was performed and using all three drugs administered, no difference in ONH blood flow and OPP response was recorded (p = 0.43 and p = 0.69, respectively).

## Conclusions

The findings of this study indicate that NO seems to be involved in basal regulation of ONH blood flow. However, this was not the case during isometric exercise. Whether different regulatory systems gain importance after increase of OPP and blood flow has to be the focus of further studies.

